# Metabolic and Flavor Dynamic Changes in *Aronia melanocarpa* Juice During Fermentation and 90-Day Storage

**DOI:** 10.3390/foods15122094

**Published:** 2026-06-10

**Authors:** Ranran Ma, Xiaotong Wu

**Affiliations:** College of Life Sciences, Inner Mongolia University, Hohhot 010070, China; 15849255352@163.com

**Keywords:** *Aronia melanocarpa*, fermentation, storage, metabolomics, flavor and antioxidant activity

## Abstract

This investigation evaluated temporal variations in metabolic fingerprints, sensory properties, and functional traits of *Aronia melanocarpa* syrup throughout the fermentation stage followed by an additional 90-day preservation interval. Findings indicated that microbial transformation notably increased the cumulative polyphenolic levels, overall flavonoid concentrations, and DPPH free radical quenching efficiencies of the preparation, which stayed consistent over this duration. Non-targeted metabolomics screening recognized a combined 1918 chemical signatures, whereby phenylpropanoids, fatty acids, and terpenes represented critical components. A total of 1591 distinct metabolites were identified, demonstrating substantial accumulation inside the flavonoid synthesis and pyruvate catabolic pathways. Furthermore, 355 volatile substances were characterized, with aldehydes and esters acting as vital elements providing honey-scented and fragrant tastes. The cooperative impacts of brewing and aging improved the nutritive and gustatory merits of *Aronia melanocarpa* syrup, providing a theoretical framework for developing superior fermented commodities.

## 1. Introduction

Categorized under the Rosaceae family, *Aronia melanocarpa* represents a deciduous shrub native to Eastern North America as well as Northeastern Canada. After being imported into Europe during the early twentieth century, this species has gained extensive propagation throughout various global areas [1]. Inside China, its main cultivation zones are primarily centered in Liaoning, Jilin, and Heilongjiang Provinces [2]. This bush generally attains dimensions ranging from around 0.5 to 1.0 m, with its berries representing the primary component consumed. The drupe possesses elevated levels of phenolic acids, anthocyanins, flavonoids, and proanthocyanidins, exhibiting polyphenol concentrations that exceed numerous different dark-hued fruits, gaining it the nickname “champion of anthocyanins” [3,4,5]. Nevertheless, the natural tartness and bitterness inherent in raw black chokeberries (tannin levels peaking at 1.894 mg/g) hinder their direct consumption [1,6]. Fermentation methods help enhance its gustatory characteristics.

Integrating metabolomics into flavoromics offers essential technological assistance for comprehensively understanding the quality transformation trajectories of preserved products. Specifically, metabolomics precisely demonstrates the metabolic shifts concerning non-volatile elements throughout aging and storage phases by identifying key functional biomarkers [7]; conversely, flavoromics explicitly tracks the temporal variations in volatile molecules to identify primary aroma components [8]. Merging these dual omic strategies permits constructing a multi-level “metabolite-flavor-function” interactive framework, which enhances the integrated assessment of nutritional benefits along with gustatory excellence for sophisticated fruit beverages [9,10].

Numerous scholarly investigations have examined utilizing lactic acid bacterial fermentation to augment the complexity of black chokeberry juice. Initially, such research indicates that integrating lactic acid species including *Lactobacillus plantarum*, *Lactobacillus acidophilus*, or *Lactobacillus rhamnosus* notably elevates total phenolic and flavonoid concentrations in concentrates, while distinctly enhancing DPPH and ABTS radical quenching strength and decreasing antioxidant fragility; moreover, structural modification and intensification of polyphenol function act as primary mechanisms fostering the reinforced antioxidative potential [11]. Variations in strain-specific efficacy exist regarding fermentative yield. For instance, employing *Lactobacillus plantarum* 1243 resulted in minor reductions in general phenolic and total flavonoid quantities [12]; yet, the reductive defense of the beverage stayed constant, whereas olfactory beauty and palatability were noticeably enhanced [12]. Furthermore, *Lactobacillus plantarum* C8-1 has likewise been applied to ferment black chokeberry syrup to generate a premium probiotic liquid with a distinctive aromatic profile [13]. Flavoromics evaluations have additionally verified that lactofermentation could efficiently eliminate objectionable volatile elements like 1-penten-3-one and propanal, enrich ester- and floral-aromatic substances including 2-pentanone and 1-butanol-3-methyl acetate, and considerably rearrange the flavor architecture of the extract [11,14]. Integrated metabolomics examination has demonstrated that fermentation profoundly modifies the distribution of organic acids, phenolic acids, and anthocyanins within juice, improves the bioaccessibility of polyphenols, and finally maximizes overall product excellence [2,12].

Prior significant research has undoubtedly proven that the quality enhancement impacts of solitary or blended Lactobacillus strains regarding black chokeberry juice are critical. Nonetheless, most of these inquiries concentrate on diverse sensory shifts prior to and following fermentation, ignoring meticulous investigation into kinetic fluctuations in nutritive factors and aromatic traits throughout prolonged aging of the inoculated mixture. Indeed, the biochemical attributes, microbial metabolites, and volatile molecules of processed berry and vegetable essences are not stable during ripening but undergo ongoing temporal rearrangement [7,15]. Researchers have verified that preserved fruit and vegetable drinks resembling bilberry, hawthorn, apricot, and quince undergo metabolomic restructuring during storage, with particular mixes attaining improved taste and health benefits, and the sequences of functional development remain highly different depending on the initial raw ingredient composition [16,17,18,19,20,21]. At present, research on the fluctuating processes of polyphenol degradation, antioxidant capacity, and fragrance progression in aged black chokeberry nectar across a 90-day extended timeframe is fairly scarce.

Subsequently, this study utilized a sequential brewing process involving *Saccharomyces cerevisiae* and *Lactobacillus plantarum* to manage black chokeberry nectar, while implementing a rigorous chilled conservation method at 4 °C for 90 days and thoroughly examining quality shifts across the whole brewing and stabilization period. Following the 90-day holding time, the probiotic drink retained stable enhanced concentrations of total phenolic compounds, total flavonoid amounts, and DPPH free radical neutralizing capacity, suggesting that its antioxidative strength stayed unchanged. Integrated metabolomics with sensory assessment revealed that the manufacturing stage mainly depended on microbially mediated catabolism to elevate the buildup of medicinal polyphenols and produce alcoholic-related and estery-scented organic molecules, whereas the extended storage period additionally triggered subsequent restructuring of materials, leading to aroma and therapeutic attributes different from those recognized during fermentation. This project aims to fully delineate how volatile odorants and non-volatile bioactive factors in black chokeberry juice fluctuate progressively through brewing and long-term preservation. A supplementary objective is to detect the intrinsic connection between metabolic reconfiguration and flavor evolution, thus setting up a conceptual and practical framework for preserving quality uniformity and enhancing high-grade application of fermented black chokeberry commodities.

## 2. Materials and Methods

### 2.1. Experimental Materials

Specimens of *Aronia melanocarpa* were collected from the Honghui Fruit and Vegetable Specialized Cooperative situated in Dalian, Liaoning Province, China. An adequate amount of distilled water was introduced, followed by blending the substance into a uniform solution using a juice press. That mixture underwent an 80 °C steam treatment for a period of 30 min. This procedure aimed at removing residual bacterial groups and inherent fungal strains, which is vital for ensuring the stability of combined experimental cultures. Finally, the purified homogenate was chilled at ambient temperature and kept for the biochemical assessments that followed.

### 2.2. Reagents and Sources

Every investigation employed premium-quality reagents with purities surpassing 95%, combined with HPLC-grade solvents exhibiting concentrations above 98%, guaranteeing the dependability and precision of those empirical results. These providers included: CNW Technologies for methanol and acetonitrile, SIGMA-ALDRICH for acetic acid, and Watsons for ultrapure water. Each designated solvent and chemical was utilized directly from its primary containers without undergoing any additional purification procedures.

### 2.3. Strain Activation and Culture

The 71B cellular population of *Saccharomyces cerevisiae* was procured from Lallemand (France). An exact gram of active yeast was precisely measured and mixed with 50 mL of filtered water, followed by cultivation within a thermoregulated environment at 30 °C for twenty minutes to produce an intensified yeast fluid.

*Lactobacillus plantarum* isolate WXD123 was supplied by the Life Sciences Division of Inner Mongolia University. Under aseptic conditions, a tiny quantity of glycerol-stored microbial cells was blended with MRS broth fluid and cultivated aerobically at 37 °C over 24 h to finish the initial activation process. Following this, that seed was diluted using an aspect ratio of 0.5% (*v*/*v*) in new MRS liquid medium and incubated further under those identical parameters for 6–8 h to acquire live cell communities throughout the logarithmic developmental stage for the fermentation trials that followed.

### 2.4. Experimental Design and Fermentation Process

This investigation developed two separate experimental cohorts, AJ (*Aronia melanocarpa* juice) and FAJ (fermented *Aronia melanocarpa* juice), incorporating six unique biological duplicates within each category. This production procedure employed the refined operational protocols identified through preceding pilot trials conducted by our academic team. The sucrose concentration inside the filtered liquid was adjusted to 12 °Brix, while the acid level stayed at 4.5, and the preparation rate was defined as a dried-to-wet ratio of 1:1. Primarily, yeast inocula were added as an activator at a volumetric proportion of 0.9% (*v*/*v*) and underwent incubation under ambient environments at 32 °C for 25 h. Following this, *Lactobacillus plantarum* strains were integrated as a probiotic agent at a volumetric ratio of 1.4% (*v*/*v*), and further maturation proceeded at 37 °C for 24 h.

### 2.5. Quantification of Total Phenolic Content

The total polyphenol content (TPC) was evaluated via a Folin–Ciocalteu spectrophotometric technique adapted by Škerget [22]. After centrifugation, the supernatant was resuspended in purified water to achieve a specific 20:1 dilution factor. One part of this diluted liquid, totaling 0.5 mL, was utilized for subsequent testing. Specifically, 0.5 mL of distilled water and 2.5 mL of 0.1 M Folin–Ciocalteu reagent were mixed thoroughly, followed by a five-minute incubation interval. Subsequently, 2 mL of 7.5% Na_2_CO_3_ was added. Following the completion of the chromogenic phase, the absorbance intensity was measured at 765 nm with a microplate reader to determine TPC according to the established standard curve y = 0.0026x + 0.0459 (R^2^ = 0.9981).

### 2.6. Quantification of Total Flavonoid Content

Regarding the computational assessment of total flavonoid quantity (TFC), the refined aluminum chloride spectrophotometric method described by Zhang [23] was utilized. All specimens and components were subjected to a volume reduction factor of tenfold before performing the optical density analysis. After the complexation step, the spectral absorbance was captured at 400 nm using a fully automated microplate detector, with the overall flavonoid level calculated through the reference calibration line y = 2.1757x + 0.0297 (R^2^ = 0.9977), where outcomes are expressed as μg RE/mL.

### 2.7. Dpph Radical Scavenging Activity

The determination procedure of DPPH radical scavenging activity was as follows. Vitamin C (Vc) was used as the positive control in this experiment, and the sample solution was prepared into a series of concentration gradients. Briefly, 2 mL of sample solution at different concentrations was added to the reaction system, followed by the addition of 0.4 mmol/L DPPH solution and thorough mixing. The mixture was kept in the dark for 30 min, and the absorbance was measured at 517 nm and recorded as A_1_. Subsequently, absolute ethanol was used to replace the DPPH working solution and the sample solution, and the absorbance values A_2_ and A_0_ were determined sequentially under the same conditions. The scavenging rate was calculated according to the following formula:

$$\mathrm{DPPH}\;\mathrm{radical}\;\mathrm{scavenging}\;\mathrm{activity}\;(\%)\;=\;(1-\frac{A_{1}-A_{2}}{A_{0}})\;\times 100.$$

### 2.8. Uplc-Ms/ms Untargeted Metabolomics Analysis

#### 2.8.1. Extract Metabolites from the Samples

An amount of 100 μL of the specimen was transferred into a centrifugal tube; subsequently, 400 μL of the extraction liquid was introduced. This mixture incorporates methanol and acetonitrile blended in a 1:1 volume proportion, featuring a specific internal marker. It was intensely vortexed for 30 s to ensure complete uniformity; then, ultrasonication within an icy hydrocarbon bath was executed for 10 min. Finally, the mixture was cooled down at −40 °C for 1 h. Following that, centrifugation was implemented at 4 °C and 12,000 rpm for 15 min. By adhering to this method, the upper organic portion is withdrawn into a vial for subsequent instrumental evaluation. At the same time, the identical quantity of the supernatant was gathered from every control batch and completely blended to create quality control specimens, which were examined concurrently with the experimental groups.

#### 2.8.2. Instrumental Analysis Conditions

To achieve metabolic profiling, an ultra-high-performance liquid chromatography system (Thermo Fisher Scientific, Waltham, MA, USA) Vanquish was integrated with a Phenomenex Kinetex C18 packing column (2.1 × 50 mm,2.6 μm). The mobile phase included component A (a 0.01% acetic acid solution in water) and component B (isopropanol blended with acetonitrile in a 1:1 volume ratio, *v*/*v*). That stationary support kept its temperature at 25 °C, the injection interface was chilled to 4 °C, and every sample size stayed uniform at 2 μL.

Metabolomic profiling utilized an Orbitrap Exploris 120 mass analyzer (Thermo Fisher Scientific, USA), whereby every sample was analyzed utilizing Xcalibur V4.4 software. For electrospray ionization, specific parameters comprised a sheath gas flow rate of 50 Arb, an auxiliary gas flow rate of 15 Arb, and a capillary drying gas flow rate of 1 Arb; the spray needle temperature was fixed at 320 °C and the heated source chamber temperature was kept stable at 350 °C. Total-ion chromatograms were recorded with a 60,000 resolving power setting, whereas fragmentation data were gathered under 15,000 resolving power conditions, employing stepped normalized collision energy (SNCE) values of 20, 30, and 40. The capillary voltage was fixed at 3.8 kV in positive-ion mode, while −3.4 kV was applied for negative-ion acquisition.

#### 2.8.3. Data Processing

After gathering empirical datasets, initial mass spectroscopic raw data was converted into mzXML structures through ProteoWizard (V3.0.24054). Then, XCMS workflows were employed to execute peak identification, retention period separation, and temporal alignment processes on refined dataset fragments. Sample maps showing missingness ratios exceeding 50% were excluded from further analytical assessments. Gaps in the spectra were filled using a k-nearest neighbor (KNN) technique, while signal intensities were standardized by means of support vector regression (SVR). Metabolomic profiling was conducted utilizing two repositories: BiotreeDB (V3.0, a comprehensive compound archive) and BT-Plant (V1.1, a plant-oriented specific database). Unique metabolites meeting the following requirements were identified for further examination: an assignment certainty level over 0.5 and a QC duplicate variability (CV) ratio less than 0.5. This research combined three QC controls with eighteen authentic specimens. Altogether, 40,190 spectral markers were derived from primary raw records, and comprehensive outcomes are documented in the supplementary raw data spreadsheet.

### 2.9. Hs-Spme-Gc-Ms Non-Targeted Volatile Metabolomics Analysis

#### 2.9.1. Spme-Based Sample Preparation and Extraction

The 20 mL container accommodated the sample; thereafter, it was treated with 10 μL of a domestic reference reagent (2-octanol stored at 10 mg/L within a liquid solution). The PAL instrument’s SPME device was configured to execute a temperature profile of 60 °C. This analyte underwent a fifteen-minute heated separation period, followed by a thirty-minute stabilization stage, and ultimately participated in a four-minute extraction procedure.

#### 2.9.2. Gc-Ms Instrumental Analysis Conditions

Regarding GC-MS evaluation, a sophisticated Agilent 7890/5977B apparatus equipped with a DB-Wax stationary phase was utilized (Agilent Technologies, Santa Clara, CA, USA). These specimens were injected utilizing a splitless technique with helium serving as the carrier gas. The velocity inside that capillary column was kept steady at 1 mL/min, while the injector purge rate was configured to be 3 mL/min. Concerning the thermal program, it consisted of: preserving 40 °C for 4 min, followed by a temperature increase of 5 °C/min reaching 245 °C, then maintaining this peak for 5 min. Precise parameters for the inlet, transfer line, source, and quadrupole were controlled at 250 °C, 250 °C, 230 °C, and 150 °C, respectively. Mass spectra (EI ion source, 70 eV) were collected in a full-scan manner in the range of *m*/*z* 20–400, incorporating a solvent delay of 2.37 min.

#### 2.9.3. Data Preprocessing and Annotation

The GC-MS results were examined using raw data via LECO Chroma TOF 4.3X software connected to the NIST mass spectrum repository. This technical approach merged peak recognition, baseline adjustment, and retention time alignment, as well as spectral deconvolution, substance differentiation, and peak area calculation, eventually executing ultimate mass spectral correlation relative to the NIST database [24].

### 2.10. Statistical Analysis

To ensure dataset homogeneity, every biological replicate was evaluated in triplicate. Quantitative metrics were analyzed utilizing Microsoft Excel 2019; where findings were expressed as mean figures alongside standard deviations (mean ± SD). Graphical representations were generated using OriginPro 2020 and GraphPad Prism 8 tools. Concerning metabolic profile classification, hierarchical clustering analysis (HCA) was executed via base R libraries within the R 4.2.1 programming framework and illustrated in a heatmap format. Applying SIMCA 18 software [25], we performed unsupervised PCA coupled with OPLS-DA to calculate subsequent VIP values. Molecular structural determination and functional pathway assignment were achieved by querying the KEGG compound database [26] and pathway network [27]. Additional data conversion and visual rendering were conducted through specific R packages.

## 3. Results

### 3.1. Changes in Total Flavonoids, Total Phenolics, and Dpph Radical Scavenging Activity

**Figure 1:**
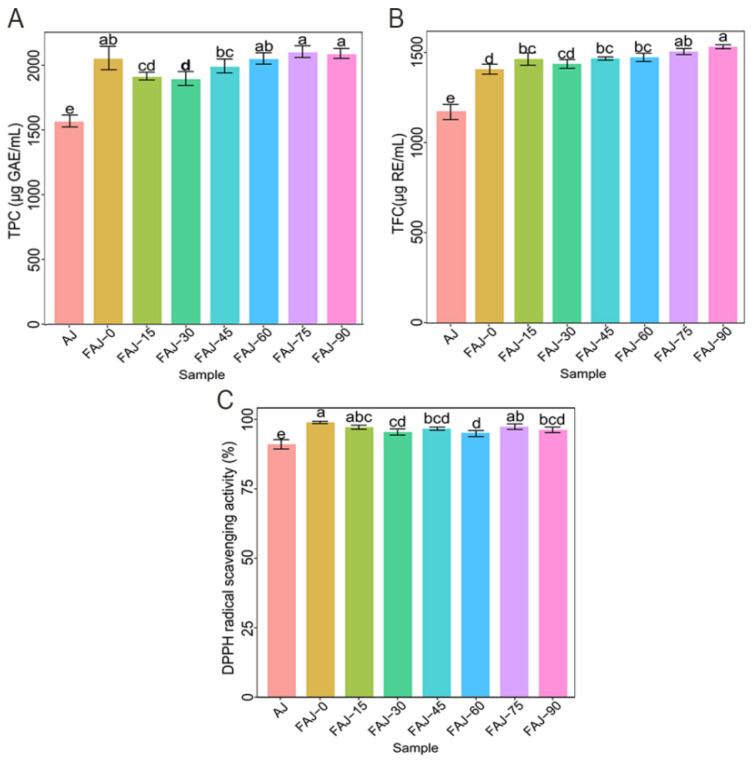
Evolution of functional elements and antioxidant potency within aronia nectar throughout enzymatic treatment and ninety-day preservation. (**A**) Aggregate polyphenol concentration (APC); (**B**) general flavonoid concentrations (AFC); (**C**) DPPH free radical inhibition power. AJ denotes non-processed aronia juice; FAJ designates fermented aronia nectar (enzymatic-based production); FAJ-0, -15, -30, -45, -60, -75, and -90 denote filtered elixir stored for 0, 15, 30, 45, 60, 75, and 90 days, accordingly. Distinct lowercase letters over every column indicate substantial variations between categories (*p* < 0.05).

Similar patterns occurred regarding overall flavonoid levels, general phenolic potency, and DPPH free radical inhibition in the black chokeberry elixir through the fermentation period and 90-day holding durations (Figure 1). After finishing processing, the aggregated flavonoid quantity within this beverage notably elevated, surging from 1169.49 μg RE/mL in the baseline specimen (AJ) to 1407.23 μg RE/mL for the FAJ-0 variant (Figure 1B). Such expansion aligns with the synergistic influence of lactobacilli and saccharomyces microbes in managing polyphenol synthesis [28]. During primary preservation stages, the environmental surroundings underwent an ongoing decrease in atmospheric oxygen tension. Simultaneously, ethanol produced by microbial-assisted anaerobic glycolysis enhanced polyphenol solubility, leading to a steady incremental rise in identified total flavonoid figures, ultimately attaining 1531.80 μg RE/mL upon the 90th day of industrial commercialization [29].

Total polyphenol contents rose markedly throughout the entire brewing process, where the FAJ-0 isolate exhibited a distinctly greater enhancement compared to its AJ sibling. This phenomenon results from the synthesis of polyphenolic compounds triggered by lactic acid bacterial cellular activities [30]. Similar trends are found in various lactobacillus-fermented products like blended fruit juices [31], concentrated mango syrup [32], and silkworm honey [33]. During the early equilibrium phase (15–30 days), total polyphenol amounts fell marginally due to enzymatic or inherent oxidative processes and phenol–protein complexes [34]; subsequently, these values consistently rebounded and sustained a raised plateau during both the middle and ultimate aging phases (45–90 days), permanently surpassing the concentrations of their unprocessed baseline counterparts (Figure 1B).

The microbial enzymatic hydrolysis notably improved the antioxidant potential of this extract. The DPPH free radical quenching activity for FAJ-0 reached peak levels at a rate of 98.89%. Analogously, fermented products like longan syrup, blended juices, and olive oil demonstrated elevated antioxidant potency [31,35,36]. During every preservation phase, although minor variations occurred in DPPH radical inhibiting values, these metrics stayed persistently over 94% and were noticeably greater than those noted in the unfermented control (Figure 1C). Such correlation originated from the massive quantities of total polyphenolic substances and complete flavonoids existing inside this blend [28].

To summarize, enhancing the concentrations of phytochemicals alongside protective properties within black chokeberry nectar depends largely on non-aerobic processing. Throughout the complete 90-day preservation interval, these nutritional advantages for the beverage stayed mostly consistent and demonstrated an extremely beneficial pattern concerning its metabolic shifts.

### 3.2. Untargeted Metabolomics

To investigate metabolic profiles and compositional changes within freshly pressed, aged, and cultured juice extracts throughout the ninety-day maturation period, this research utilized liquid chromatography–mass spectrometry (LC-MS)-driven non-targeted metabolomics. Quality control (QC) protocols are vital for guaranteeing the authenticity and rigorous accuracy of metabolomic information. As displayed in the PCA score graph for QC specimens (Figure S1), the compact grouping of every QC sample validated the consistent analytical performance, superior dataset integration, and adequate reproducibility alongside the replicability of the utilized approach.

**Figure 2:**
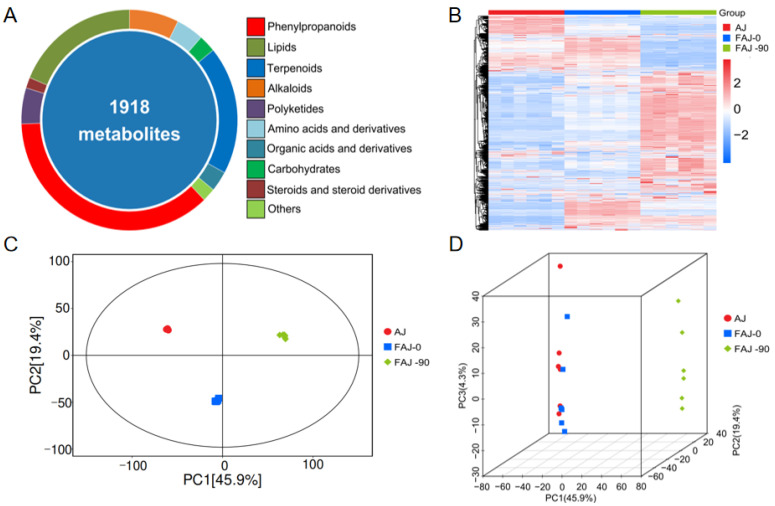
An exhaustive evaluation of metabolites across three specific clinical cohorts: (**A**) counting of identified chemical species; (**B**) a vertical clustering heatmap integrated with a dendrograph illustrating metabolic signatures; (**C**) two-dimensional PCA projection diagram; (**D**) three-dimensional PCA spatial rendering. AJ, unprepared *Aronia melanocarpa* juice (baseline); FAJ-0, mature transformed *Aronia melanocarpa* nectar during the initial period; FAJ-90, long-term preserved aged *Aronia melanocarpa* nectar kept for ninety days.

By employing electrospray ionization (ESI) methods in both polar modes, we identified a total of 1918 unique metabolites in these three fruit beverage specimens (Table S1). Specifically, 971 entities were observed during positive-ion operations, while 947 were recognized in negative-ion regimes. Through systematic hierarchical categorization, it became evident that these substances comprised ten major categories and fifty-one further subgroups (Figure 2A, Figure S2), wherein phenylpropanoids (36.60%), lipids (18.82%), and terpenoids (18.72%) predominated as essential metabolic elements. Subsequently, hierarchical clustering coupled with principal component analysis (PCA) was utilized to assess sample consistency and intrinsic chemical disparities. Dendrogram data revealed that the metabolic patterns of individual groups remained distinctly identifiable (Figure 2B). Based on the PCA findings (Figure 2C,D), PC1, PC2, and PC3 represented 37.52%, 16.91%, and 4.3% of the overall variance, respectively. The score plots demonstrated concentrated clustering for all six biological duplicates within each group, followed by distinct separation between different experimental batches. Generally, this proof verified that the fermentation stage substantially modified the metabolomic profiles of these juices.

### 3.3. Differential Metabolite Analysis

Incorporating feature selection enhances the accuracy of OPLS-DA models by removing non-informative variables, thereby facilitating more distinct visual representations of sample separations. This study uncovered specific discriminatory patterns in each pairwise analysis, whereby six replicate samples aggregated closely within their corresponding groups, supplying additional proof regarding substantial metabolic differences among various molecular types (Figure S3A). Given that both R^2^Y and Q^2^ metrics surpassed 0.95, such architectural structures exhibited superior efficiency and stability. Furthermore, permutation validation suggested minimal chances of analytical overfitting, validating the following identification of modified metabolites throughout identified pathological populations (Figure S3B,C).

**Figure 3:**
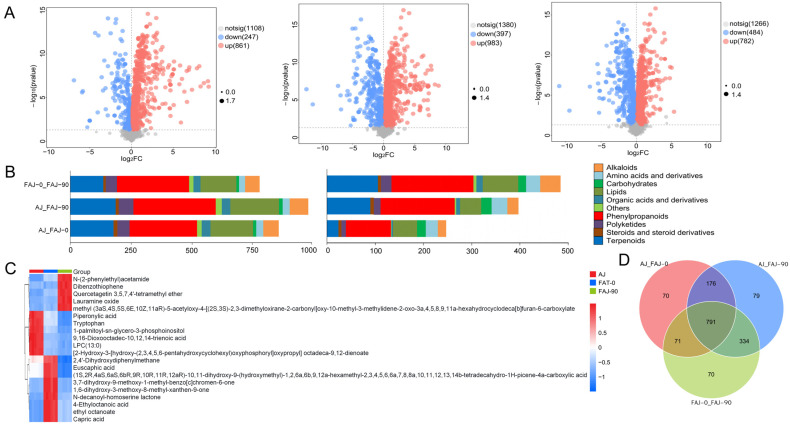
Evaluation of metabolic profiles diverged between diverse groups. (**A**) Volcano maps showing shifted metabolites, structured from left to right: AJ versus FAJ-0, AJ versus FAJ-90, and FAJ-0 versus FAJ-90. (**B**) Categorization of disrupted metabolites, displaying increased elements on the left together with decreased ones on the right. (**C**) Dendrograph-focused heatmap concerning those two hundred most significant altered metabolites. (**D**) Venn chart depicting common modified metabolites throughout each pair of analyses. AJ, crude *Aronia melanocarpa* juice (original); FAJ-0, fresh fermented *Aronia melanocarpa* juice on day zero; FAJ-90, ripe fermented *Aronia melanocarpa* juice preserved for ninety days.

By employing the OPLS-DA approach, we determined candidate biomarkers via evaluating variable importance in projection (VIP) scores for comprehensive evaluation [37]. Utilizing a VIP cut-off exceeding 1 and *p* < 0.05 as standards, distinctly modulated metabolites were recognized in every dual comparison among these three specific clinical subsets. Regarding the particular trios—AJ vs. FAJ-0, AJ vs. FAJ-90, and FAJ-0 vs. FAJ-90—1108, 1380, and 1266 standardized metabolic entities were measured, respectively (Tables S2–S5). Variations in those molecules are displayed graphically through volcano diagrams (Figure 3A). Additional categorization of the increased and decreased metabolites during those three group contrasts sorted them into ten primary clusters. More precisely, phenylpropanoids constituted the main type inside both raised and lowered metabolites, followed by lipids and terpenoids (Figure 3B). Hierarchical grouping of the twenty most markedly altered metabolites indicated that the metabolic patterns of AJ, FAJ-0, and FAJ-90 specimens showed evident inner arrangement and outer distinction, with diverse molecules showing significant positive or negative transitions within the arrays, clearly illustrating major metabolic shifts throughout the three cohorts (Figure 3C, Table S6).

By utilizing the three specific dual comparisons established within this present study, a total of 1591 distinctly regulated metabolites were discovered, among which 791 stayed consistent throughout these three categories (Figure 3D). Utilizing K-means grouping techniques partitioned the fluctuation patterns of such compounds between these three cohorts into nine different clusters, effectively confirming that lactic acid-based metabolic pathways during subsequent storage phases imposed major influences on chemical transformations and performances (Figure S4).

To clarify the metabolic pathways governing aromatic development and functional implications within fruit beverages through ethanol fermentation and 90-day preservation stages, this work comprehensively identified divergent metabolites and constructed a regulatory framework covering various chemical groups. Utilizing lipid turnover as a model, alpha-Linolenic acid demonstrated a persistent decline throughout alcoholic conversion, whereas 9,10,18-Trihydroxystearic acid increased gradually throughout the entire period. Prior research indicated that lactic bacteria possess the potential to transform polyunsaturated fats into trihydroxy products [38], involving particular fatty acid hydratase (FA-HY1), which allows the restructuring of alpha-Linolenic acid into mono-hydroxy precursors [39]. Consequently, it is proposed that this route might be activated by the FA-HY1 enzyme of *Lactobacillus plantarum* to induce primary hydroxylation; however, the precise enzymatic process for the subsequent modification of mono-hydroxy intermediates into trihydroxy fatty acids remains undefined and requires additional meticulous exploration. In addition to lipids, flavonoids likewise experienced major architectural shifts. Results indicate that glycosylation of flavonols might markedly boost the aqueous solubility and durability of these molecules, a procedure coordinated by glycosyltransferases sourced from plants, microbes, or fungi [40,41]. This investigation observed that during brewing and extended storage, the levels of certain flavonoid monoglycosides consistently declined, while disaccharide or polysaccharide flavonoids like Rutin, Quercetin 3-gentiobioside, and Leucoside were noticeably boosted, implying that microbial O-glycosyltransferases likely perform the O-glycosylation remodeling of flavonoids, a shift that may efficiently upgrade the pharmacological utility of flavonoids [40].

Throughout this procedure, unique amino acids like Tryptophan were consumed by microbial organisms and experienced transformations through lactylation and acetylation, causing a significant reduction in their concentrations; conversely, metabolites like N-Lactoylphenylalanine and N-Acetylphenylalanine rose sharply, indicating vigorous amino acid reshaping and encouraging the creation of flavor compounds [42]. Within anaerobic or oxygen-limited fermentation settings, microbial metabolic patterns transitioned from the tricarboxylic acid (TCA) cycle toward fermentative routes, generating reduced quantities of intermediates like Malic acid and Pyruvate. Concurrently, malolactic fermentation conducted by lactic acid bacteria additionally helped in exhausting malic acid [43,44]. The rapid breakdown of branched-chain and aromatic amino acids led to the accumulation of organic acids such as 2-Hydroxy-3-methylbutyric acid and 3-(3-Methoxyphenyl)propanoic acid [34]. The shifting utilization of sugars by microbes and polysaccharide hydrolysis similarly prompted the concentration of Galactose to initially drop prior to rising again [45] (Table S1). Furthermore, the flavonoids and phenolic acids that appeared provide varied therapeutic advantages, encompassing antioxidant, anti-inflammatory, cardiovascular-defensive, antimicrobial, and metabolic-controlling impacts [46]. Similarly, amino acid derivatives and specific organic acids boost both the sensory qualities and nutritional value of the beverage [47].

**Figure 4:**
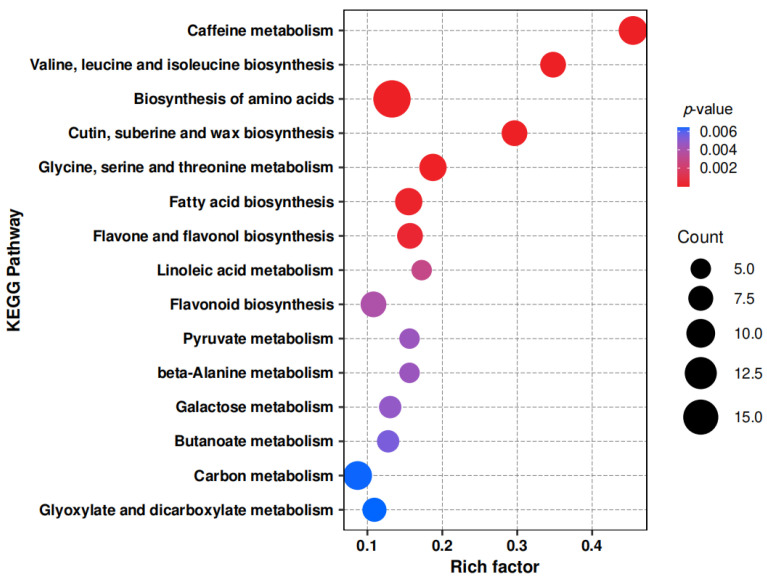
Analytical evaluation of KEGG metabolic routes concerning observed modifications in metabolic datasets. The density level of each round symbol denotes the unique *p*-values for pathways (higher shades represent lower significance, whereas brighter hues denote higher significance); concurrently, the scale of every tag reflects the total quantity of metabolites located in that specific route. The horizontal axis illustrates the Rich factor, whilst the vertical axis offers the precise titles of the recognized KEGG pathways.

The biochemical framework supporting these unique transformations was additionally clarified via KEGG pathway enrichment evaluations. Such altered metabolites exhibited overrepresentation within some pathways, including flavone and flavonol biosynthesis, flavanoid production, butanoate degradation, pyruvate metabolism, and fatty acid synthesis (Figure 4). The elevation in overall polyphenolic and collective flavonoid levels mainly resulted from the flavone and flavonol synthesis route. The transformation of particular amino acids into phenolic substances and flavonoids was intimately linked to the activation of the flavonoid biosynthetic chain. Furthermore, the presence of pyruvate metabolism and butanoate catabolism systems offered a biochemically grounded rationale for the buildup of organic acids and the creation of aromatic esters.

### 3.4. Differential Analysis of Volatile Compounds

**Figure 5:**
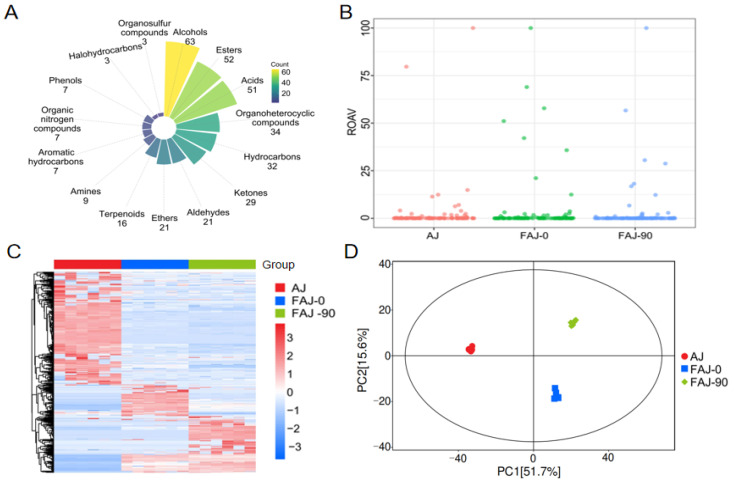
Evaluation of aromatic patterns within three unique categories of *Aronia melanocarpa* juice beverages. (**A**) Pie diagram demonstrating the proportional distribution of volatile substances; (**B**) graph presenting relative odor activity values (ROAV) for flavoring constituents; (**C**) hierarchical clustering heatmap regarding fragrance molecules; (**D**) principal component analysis (PCA) distribution chart for volatile segments. AJ, untreated *Aronia melanocarpa* nectar (baseline); FAJ-0, aged fermented *Aronia melanocarpa* juice collected on initial day; FAJ-90, fermented *Aronia melanocarpa* juice kept for 90 days.

To investigate the volatile organic substances present in these three unique sample groups, gas chromatography integrated with mass spectrometry served as the primary analytical tool. Altogether, 533 volatile components were identified, including 178 newly discovered constituents; however, 355 were grouped into fifteen major chemical classes. Alcohols constituted the predominant group, followed by esters and carboxylic acids (Figure 5A, Table S7). To evaluate the precise impact of specific volatiles on sensory properties (Figure 5B), we computed the relative odor activity values (ROAVs) and created an appropriate scatter diagram. The ROAV of the most abundant molecule regarding total aroma was normalized to 100 for comparative reasons. Hierarchical clustering coupled with principal component analysis (PCA) demonstrated that volatile profiles across those three groups were distinctly separated, with every six biological replicate per group closely bunching on score plots while exhibiting substantial inter-group differences (Figure 5C,D). Partial least squares-discriminant analysis (PLS-DA) highlighted distinct segregational tendencies between every pair of groups, subsequently resulting in tight grouping of specimens within their respective categories (Figure S5). These findings imply that volatile composition frameworks varied markedly among diverse experimental batches. Generally, such data verified that both fermentation phases and storage conditions profoundly altered the volatile metabolic features of the juice.

**Figure 6:**
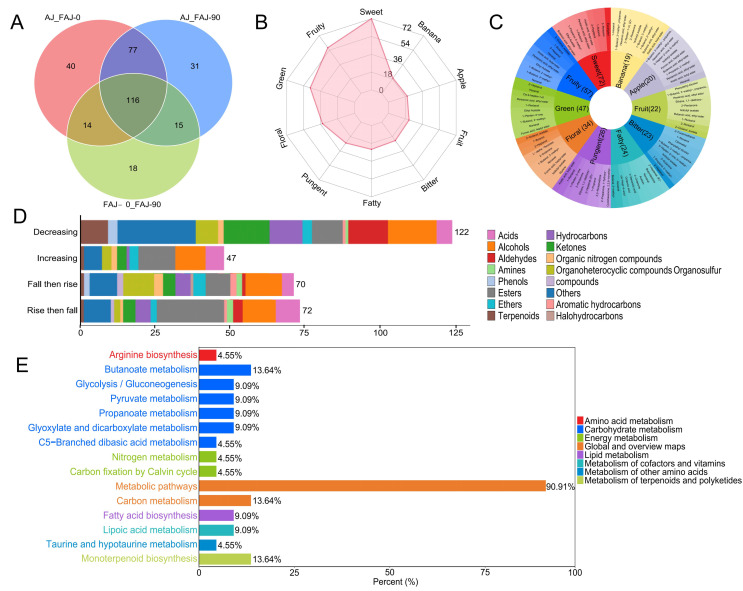
Comprehensive evaluation of varied aromatic constituents within *Aronia melanocarpa* nectar. (**A**) Venn plot illustrating exclusive volatile compounds; (**B**) radar graph representing sensory olfactory traits; (**C**) pie chart categorizing odor-linked molecules; (**D**) cluster heatmap presenting variable volatile ingredients; (**E**) bar graph demonstrating KEGG pathway examination results regarding altered volatile components. AJ, unprocessed *Aronia melanocarpa* juice (baseline); FAJ-0, freshly obtained *Aronia melanocarpa* juice gathered on day 0; FAJ-90, fermented *Aronia melanocarpa* juice preserved for 90 days.

Concerning the comprehensive tri-partite evaluation, 311 distinctive volatile metabolites were identified and categorized, encompassing 116 common among these three groups (Figure 6A). Architectural examination indicated that 122 altered compounds consistently declined throughout both developmental and storage phases, whereas 47 continually rose. Furthermore, 72 variables demonstrated a predominant increase followed by a subsequent reduction, while 70 manifested an early drop before final recovery (Figure 6B).

Every individual volatile substance contributed uniquely to the overall fragrance of this beverage. Analysis through a sensory chart revealed that sugary, fruity, herbal, and perfumy notes comprised the primary components of the entire bouquet. Furthermore, fatty, piercing, pear-esque, and banana-related tones were likewise essential, collectively illustrating a profound, intricate, and multifaceted perceptual atmosphere (Figure 6C).

Esters and ethyl derivatives serve as vital constituents defining the distinctive olfactory signature of aged spirits [48]; these substances demonstrated specific inverse fluctuating patterns during the complete 90-day aging process following fermentation. At first, concentrations of higher alcohols, including 2-methyl-1-propanol, 1-butanol, 2-heptanol, 1-pentanol, and 3-pentanol, gradually rose, contributing a sophisticated fruity, sugary, and fragrant character to the medium. Such evolution was strongly linked with fundamental substrate transformations documented earlier; the major decline in soluble sugars allowed abundant energy resources for the production of complex alcohols via the glycolytic mechanism [49]. Simultaneously, esters consisting of ethyl acetate, 3-methylbutyl propanoate, ethyl butanoate, ethyl pentanoate, and ethyl hexanoate progressively aggregated. The hydrolysis of amino acids during brewing furnished raw materials for ester formation [49], and under the influence of alcohol acyltransferases, condensation events took place between alcohols and acyl-CoAs, finally generating distinct flavor precursors [50], markedly boosting the fruity and gustatory attributes (particularly apple-like and banana-like scents) of the blend, and collectively setting up the foundation for sensory qualities. On the other hand, alcohols such as 1-hexanol and (Z)-2-penten-1-ol continuously declined, efficiently minimizing greenish, earthy, solventic, and woody notes. Methyl benzoate, ethyl benzoate, and methyl mandelate similarly slowly vanished, aiding in eliminating sharp artificial off-tastes (Figure 5D).

Decreases in specific alcohols mainly resulted from their utilization through esterification processes. Conversely, the persistent buildup of esters throughout the ultimate maturation phase was fueled by a plentiful supply of reactants, particularly alcohols and carboxylic acids. Carboxylic acids generally showed a primary rise followed by a decline, with a minor group demonstrating steady growth. Acetic acid, hexanoic acid, and pentanoic acid maintained growing trends, providing refreshing acidity and viscous textures, while functioning as crucial elements for ester formation and enhancing the creation of distinctive esters like ethyl acetate, heptyl formate, and 3-methylbutyl propanoate, thereby intensifying the potency of fruity scents [51]. Octanoic acid and n-decanoic acid started rising prior to dropping, which avoided overly intense sour notes (Figure 5D).

Numerous aromatic components within this structure exerted collaborative control via an “integration-disappearance” enzymatic route [52]. Fatty aldehyde and organic acid motifs generated esters during bacterial processes, whereas certain esters underwent cleavage to generate various types of intermediates. Linear aldehyde substances (hexanal, nonanal) added green and buttery notes, ketones (2-pentanone and 6-methyl-5-hepten-2-one) offered sweetness together with mild spicy scents, terpenes (eucalyptol, α-terpineol, and citronellol) brought floral and fragrant attributes, and lactones (γ-butyrolactone) added a delicate bouquet (Figure 6D, Table S8).

Supplementary KEGG pathway analyses identified the biochemical underpinnings of the identified alterations. These perturbed metabolites were mainly distributed throughout diverse networks, including monoterpenoid biosynthesis, butanoate degradation, carbon cycles, glycolysis/glucogenesis, pyruvate metabolism, propanoate catabolism, and fatty acid production (Figure 6E). Notably, glycolytic/glucogenic pathways linked to pyruvate remodeling supplied substrates for alcoholic fermentation. Simultaneously, fatty acid biogenesis and butanoate metabolism were intimately tied to the buildup of different organic acids. Furthermore, participation in monoterpenoid formation accounted for the role of terpenoids in generating sweet and aromatic qualities. This transition between distinct substances constructed a multifaceted flavor biosynthetic framework (Figure S6, Table S9), collectively mapping out the gustatory profile of the produced beverage characterized by intense fragrance, diminished piquancy, and general equilibrium.

## 4. Conclusions

This current research clarified the temporal variations in practical components and aromatic compounds within black currant juice throughout a three-month shelf-life duration. Subsequent step-wise fermentation utilizing yeast and *Lactobacillus plantarum* was followed by sterilization, and this concoction demonstrated notably enhanced antiradical potency and sensorial attributes. Such enhancement resulted primarily from shifts in phenolic synthesis and pyruvic acid transformation routes, combined with biological processes involving lipid hydrolysis and flavonoid modification. These results offer a conceptual framework for commercial manufacturing and quality conservation of probiotic berry drinks.

## 5. Limitations and Future Perspectives

Several intrinsic constraints exist within this existing investigation. Although our assessment recognized various metabolic routes according to identified modifications in metabolites, the precise functional states of essential enzymes were not verified through empirical experiments. Subsequent inquiries should integrate enzymatic reactivity mapping or proteomic techniques for enhanced certainty. Throughout the trial, Lactobacillus and Saccharomyces genera were co-incubated, obstructing an accurate differentiation of microbiotic derivatives generated by each individual strain separately. Such distinctions might be elucidated in following investigations utilizing particular strain-specific benchmarks. The incubation and preservation protocols remained stable (4 °C, 90 days); however, the impact of marginal factors like pH levels, thermal conditions, and atmospheric stress was ignored during structured evaluations. Moreover, the 90-day duration solely demonstrates transient to mid-term qualitative shifts. Furthermore, this project was executed at the laboratory level; future pilot-scale tests would more thoroughly assess the functional feasibility of these results. These hurdles shall be progressively mitigated through upcoming academic pursuits.

## Data Availability

The primary findings reported within this research are integrated into the principal manuscript and additional supporting materials. Any following inquiries should be addressed to the designated corresponding investigator.

## Supplementary Materials

The following supporting information can be downloaded at https://www.mdpi.com/article/10.3390/foods15122094/s1, Supplementary File S1: Figure S1. Principal component analysis (PCA) diagrams illustrating variations in metabolite compositions throughout the processing and preservation phases of Aronia nectar; Figure S2. Classification of identified substances; Figure S3. Orthogonal partial least squares-discriminant analysis (OPLS-DA) frameworks for metabolic signatures among various categories; Figure S4. Spatial–temporal patterns of notably modified metabolites; Figure S5. OPLS-DA structures concerning volatile elements existing within three kinds of *Aronia melanocarpa* drinks; Figure S6. Consolidated paradigm incorporating distinct metabolites, biochemical routes and metabolic systems. Supplementary File S2: Table S1. Comparative proportions of metabolic factors across three unique clinical categories; Table S2. Summary of perturbed metabolite distributions in each individual contrasting group; Table S3. Elevated (red) or diminished (blue) metabolites between AJ and FAJ-0 populations; Table S4. Enhanced (red) vs. reduced (blue) metabolites within AJ versus FAJ-90 cohorts; Table S5. Amplified (red) or suppressed (blue) metabolites between FAJ-0 and FAJ-90 intervals; Table S6. Quantitative evaluation of the twenty primary substantial changed metabolites; Table S7. Analytical characterization of volatile compounds; Table S8. Flavor wheel interpretation for varied volatile molecules; Table S9. Data overview regarding metabolic pathway network architectures.

## Abbreviations

AJ	*Aronia melanocarpa* Juice
FAJ	Fermented *Aronia melanocarpa* Juice
QC	Quality Control
TPC	Total Phenolic Content
TFC	Total Flavonoid Content
DPPH	2,2-Diphenyl-1-picrylhydrazyl
RE	Rutin Equivalent
Vc	Vitamin C
GC	Gas Chromatography
MS	Mass Spectrometry
EI	Electron Ionization
ESI	Electrospray Ionization
SNCE	Stepped Normalized Collision Energy
PAL	Precision Auto Sampler
PCA	Principal Component Analysis
OPLS-DA	Orthogonal Partial Least Squares-Discriminant Analysis
PLS-DA	Partial Least Squares-Discriminant Analysis
HCA	Hierarchical Clustering Analysis
VIP	Variable Importance in Projection
ROAV	Relative Odor Activity Value
CV	Coefficient of Variation
KNN	k-Nearest Neighbor
SVR	Support Vector Regression
KEGG	Kyoto Encyclopedia of Genes and Genomes
NIST	National Institute of Standards and Technology
MRS	De Man, Rogosa and Sharpe medium
TCA	Tricarboxylic Acid cycle
FA-HY1	Fatty Acid Hydratase 1
SD	Standard Deviation
*v*/*v*	Volume per Volume

## References

[1] Ren Y., Frank T., Meyer G., Lei J., Grebenc J.R., Slaughter R., Gao Y.G., Kinghorn A.D. (2022). Potential Benefits of Black Chokeberry (*Aronia melanocarpa*) Fruits and Their Constituents in Improving Human Health. Molecules.

[2] Li X., Wang W., Li Y., Ma F., Duan C., Li D. (2026). Deciphering flavor profile and metabolic pathways in black chokeberry co-fermented by Lactiplantibacillus plantarum and Hansenula sp. via integrated volatilomics and metabolomics. Food Res. Int..

[3] Denev P., Číž M., Kratchanova M., Blazheva D. (2019). Black chokeberry (*Aronia melanocarpa*) polyphenols reveal different antioxidant, antimicrobial and neutrophil-modulating activities. Food Chem..

[4] Frumuzachi O., Rohn S., Mocan A. (2024). Fermented black chokeberry (*Aronia melanocarpa* (Michx.) Elliott) products—A systematic review on the composition and current scientific evidence of possible health benefits. Food Res. Int..

[5] Wang J., Klein C., Cochran J.R., Sockolosky J., Lippow S.M. (2025). Exploring new frontiers in LAG-3 biology and therapeutics. Trends Pharmacol. Sci..

[6] Witczak T., Stępień A., Gumul D., Witczak M., Fiutak G., Zięba T. (2021). The influence of the extrusion process on the nutritional composition, physical properties and storage stability of black chokeberry pomaces. Food Chem..

[7] Ruan W., Liu J., Guo H., Yang S., Niu M., Yu H., Meng X. (2026). From aroma to off-flavor: Metabolomics unveils the metabolic double-sided nature of traditional Chinese fermented foods. Food Chem..

[8] Yuan Y.-H., Mu D.-D., Guo L., Wu X.-F., Chen X.-S., Li X.-J. (2024). From flavor to function: A review of fermented fruit drinks, their microbial profiles and health benefits. Food Res. Int..

[9] Li X., Ma J., Chu Y., Li H., Zhang Y., Li A., Jia Y. (2025). Multi-Omics Elucidation of Flavor Characteristics in Compound Fermented Beverages Based on Flavoromics and Metabolomics. Foods.

[10] Zhao Y., Tong C., Gu C., Xu Q., Yao W., Qian H., Cheng Y. (2025). Untargeted flavoromics and correlation analysis reveal microbial interactions driving flavor in LAB co-fermented Beita juice. Int. J. Food Microbiol..

[11] Wang J., Wei B., Xu J., Jiang H., Xu Y., Wang C. (2024). Influence of lactic acid fermentation on the phenolic profile, antioxidant activities, and volatile compounds of black chokeberry (*Aronia melanocarpa*) juice. J. Food Sci..

[12] Liu M., Fang Y., Chen R., Cai M., Yang X., Fang Z., Fang X., Dong S. (2025). Effect of Lactobacillus plantarum 1243 fermentation on quality properties and metabolome of *Aronia melanocarpa* (Michx.) elliott juice. Food Chem. X.

[13] Zhu R., Fang Y., Li H., Liu Y., Wei J., Zhang S., Wang L., Fan R., Wang L., Li S. (2023). Psychobiotic Lactobacillus plantarum JYLP-326 relieves anxiety, depression, and insomnia symptoms in test anxious college via modulating the gut microbiota and its metabolism. Front. Immunol..

[14] Bontsidis C., Mallouchos A., Terpou A., Nikolaou A., Batra G., Mantzourani I., Alexopoulos A., Plessas S. (2021). Microbiological and Chemical Properties of Chokeberry Juice Fermented by Novel Lactic Acid Bacteria with Potential Probiotic Properties during Fermentation at 4 °C for 4 Weeks. Foods.

[15] Wei L., Van Beeck W., Hanlon M., DiCaprio E., Marco M.L. (2025). Lacto-Fermented Fruits and Vegetables: Bioactive Components and Effects on Human Health. Annu. Rev. Food Sci. Technol..

[16] Jiang K.-L., Liu L., Pan W.-J. (2025). Two lactic acid bacteria strains isolated from naturally fermented foods improves physicochemical quality, antioxidant capacity, shelf life stability and metabolic profiles of Dangshan pear (*Pyrus* spp.) juice. Food Res. Int..

[17] Han Q., Liu J., Nawaz M., Liu H., Zhang Q., Lv Z., Chen D., Yang W., Jiao Z. (2026). Changes in nutritional and volatile composition of peach puree as affected by fermentation with different Lactobacillus strains and subsequent cold storage. Food Chem. X.

[18] Lugo-Zarate L., Delgado-Olivares L., Cruz-Cansino N.d.S., González-Olivares L.G., Castrejón-Jiménez N.S., Estrada-Luna D., Jiménez-Osorio A.S. (2024). Blackberry Juice Fermented with Two Consortia of Lactic Acid Bacteria and Isolated Whey: Physicochemical and Antioxidant Properties during Storage. Int. J. Mol. Sci..

[19] Huang H., Wang Y., Guo Y.X., Tao W.J., Jia X.W., Xu Y. (2016). Antioxidant activity of *Aronia melanocarpa* enzyme. Sci. Technol. Food Ind..

[20] Yang J., Sun Y., Gao T., Wu Y., Sun H., Zhu Q., Liu C., Zhou C., Han Y., Tao Y. (2022). Fermentation and Storage Characteristics of "Fuji" Apple Juice Using Lactobacillus acidophilus, Lactobacillus casei and Lactobacillus plantarum: Microbial Growth, Metabolism of Bioactives and in vitro Bioactivities. Front. Nutr..

[21] Liao M., Han C., Liu W., Li Y.L., Chen C.L., Yuan H.Y., Zhou Y., Li K., Li H.J. (2025). Effects of Lactobacillus plantarum on physicochemical properties and volatile flavor substances of blueberry juice during fermentation and storage. Sci. Technol. Food Ind..

[22] Škerget M., Kotnik P., Hadolin M., Hraš A.R., Simonič M., Knez Ž. (2005). Phenols, proanthocyanidins, flavones and flavonols in some plant materials and their antioxidant activities. Food Chem..

[23] Shraim A.M., Ahmed T.A., Rahman M.M., Hijji Y.M. (2021). Determination of total flavonoid content by aluminum chloride assay: A critical evaluation. LWT.

[24] Kind T., Wohlgemuth G., Lee D.Y., Lu Y., Palazoglu M., Shahbaz S., Fiehn O. (2009). FiehnLib: Mass spectral and retention index libraries for metabolomics based on quadrupole and time-of-flight gas chromatography/mass spectrometry. Anal. Chem..

[25] Avohou T.H., Sacré P.-Y., Hamla S., Lebrun P., Hubert P., Ziemons É. (2022). Optimizing the soft independent modeling of class analogy (SIMCA) using statistical prediction regions. Anal. Chim. Acta.

[26] KEGG COMPOUND Database. https://www.kegg.jp/kegg/compound/.

[27] KEGG PATHWAY Database. https://www.kegg.jp/kegg/pathway.html.

[28] Li Q., Wang Y., Li J., Zhao N., Hu K., Ao X., Chen S., Yang Y., Liu S., Liu A. (2025). Unrevealing the characteristics of low-alcohol citrus juice cofermented with different lactic acid bacteria and Hanseniaspora uvarum. Food Chem. X.

[29] Liang S., Wang F., Granato D., Zhong X., Xiao A.-F., Ye Q., Li L., Zou C., Yin J.-F., Xu Y.-Q. (2023). Effect of β-glucosidase on the aroma of liquid-fermented black tea juice as an ingredient for tea-based beverages. Food Chem..

[30] Zhao J., Zhao F.Y., Shen X., Gao G.Q., Sun Z.H. (2023). Research progress on antioxidant activity and application of lactic acid bacteria. Biotechnol. Bull..

[31] Zhang H., Ma Y. (2019). Changes in quality of mixed blueberry and blackberry juice fermented by compound lactic acid bacteria. Mod. Food Sci. Technol..

[32] Liu Q.D., Hu K., Chen Y.S., Peng D., Xie B.Q., Sun Z.D. (2019). Changes of physicochemical properties during processing and fermentation of probiotic mango beverage. J. Huazhong Agric. Univ..

[33] Kwaw E., Ma Y., Tchabo W., Apaliya M.T., Wu M., Sackey A.S., Xiao L., Tahir H.E. (2018). Effect of Lactobacillus strains on phenolic profile, color attributes and antioxidant activities of lactic-acid-fermented mulberry juice. Food Chem..

[34] Leonard W., Zhang P., Ying D., Adhikari B., Fang Z. (2021). Fermentation transforms the phenolic profiles and bioactivities of plant-based foods. Biotechnol. Adv..

[35] Zhang Y., Cheng S.M., Chang C., Zhao Y.Y., Wu Z.Y., Zhang W.X. (2019). Study on the brewing technology of vinegar from olive fruit juice. China Condiment.

[36] Zou Y., Zou B. (2019). Effects of co-fermentation with yeast and lactic acid bacteria on the quality of litchi juice. Mod. Food Sci. Technol..

[37] Saccenti E., Hoefsloot H.C.J., Smilde A.K., Westerhuis J.A., Hendriks M.M.W.B. (2014). Reflections on univariate and multivariate analysis of metabolomics data. Metabolomics.

[38] Garofalo G., Farina V., Settanni L. Beyond fatty acids: New insights into bacterial fatty acid derivatives using avocado fermentation as a model system. Proceedings of the International Conference on Fermented Foods.

[39] Hirata A., Kishino S., Park S.-B., Takeuchi M., Kitamura N., Ogawa J. (2015). A novel unsaturated fatty acid hydratase toward C16 to C22 fatty acids from Lactobacillus acidophilus. J. Lipid Res..

[40] Xiao J., Muzashvili T.S., Georgiev M.I. (2014). Advances in the biotechnological glycosylation of valuable flavonoids. Biotechnol. Adv..

[41] Ma Y., Ren J., Yin W.-B., Liu X., Li W. (2025). Substrate promiscuity catalyzed by an O-glycosyltransferase MrOGT2 from Metarhizium robertsii. Mycology.

[42] Lu X., Yang C., Yang Y., Peng B. (2023). Analysis of the Formation of Characteristic Aroma Compounds by Amino Acid Metabolic Pathways during Fermentation with Saccharomyces cerevisiae. Molecules.

[43] Behringer K.I., Kapeluch J., Fischer A., Hellwig M. (2024). Metabolization of Free Oxidized Aromatic Amino Acids by Saccharomyces cerevisiae. J. Agric. Food Chem..

[44] Fahimi N., Brandam C., Taillandier P. (2014). A mathematical model of the link between growth and L-malic acid consumption for five strains of Oenococcus oeni. World J. Microbiol. Biotechnol..

[45] Huang G., Su D., Lee Y.-K., Zou X., Dong L., Deng M., Zhang R., Huang F., Zhang M. (2025). Accumulation of Water-Soluble Polysaccharides during Lychee Pulp Fermentation with Lactiplantibacillus plantarum Involves Endoglucanase Expression. J. Agric. Food Chem..

[46] Jomova K., Alomar S.Y., Valko R., Liska J., Nepovimova E., Kuca K., Valko M. (2025). Flavonoids and their role in oxidative stress, inflammation, and human diseases. Chem. Biol. Interact..

[47] Wei J., Xian P., Huang Y. (2026). Amino acids in yeast fermentation: A review of their roles from nutrients to modulators. Int. J. Food Microbiol..

[48] Yu C., Liu Y., Na Y., Wu X. (2025). Chemical and flavour dynamics in *Cyperus esculentus* L. pomace-raspberry composite fruit wine fermentation: A combined UHPLC-OE-MS and HS-SPME-GC-MS approach. Food Chem. X.

[49] José F., Angela M., Pilar H. (2009). Changes in the aromatic composition of the Vitis vinifera grape Muscat Hamburg during ripening. Food Chem..

[50] Xie X., Hu X., Shi X., Li M., Zhang Y., Lan W. (2022). Research progress on influencing factors of fruit wine aroma formation. J. Fuyang Norm. Univ. (Nat. Sci. Ed.).

[51] He Y., Wang X., Li P., Lv Y., Nan H., Wen L., Wang Z. (2023). Research progress of wine aroma components: A critical review. Food Chem..

[52] Liu S., Lou Y., Li Y., Zhao Y., Laaksonen O., Li P., Zhang J., Battino M., Yang B., Gu Q. (2023). Aroma characteristics of volatile compounds brought by variations in microbes in winemaking. Food Chem..

