# Supplementary material for: Metabolic and Flavor Dynamic Changes in Aronia melanocarpa Juice During Fermentation and 90-Day Storage
Source: Foods. 2026 Jun 10;15(12):2094. doi: 10.3390/foods15122094 (PMC13297830; doi:10.3390/foods15122094)
Supplement: Supplementary file 1 [file foods-15-02094-s001.zip › Supplementary File 1.pdf]

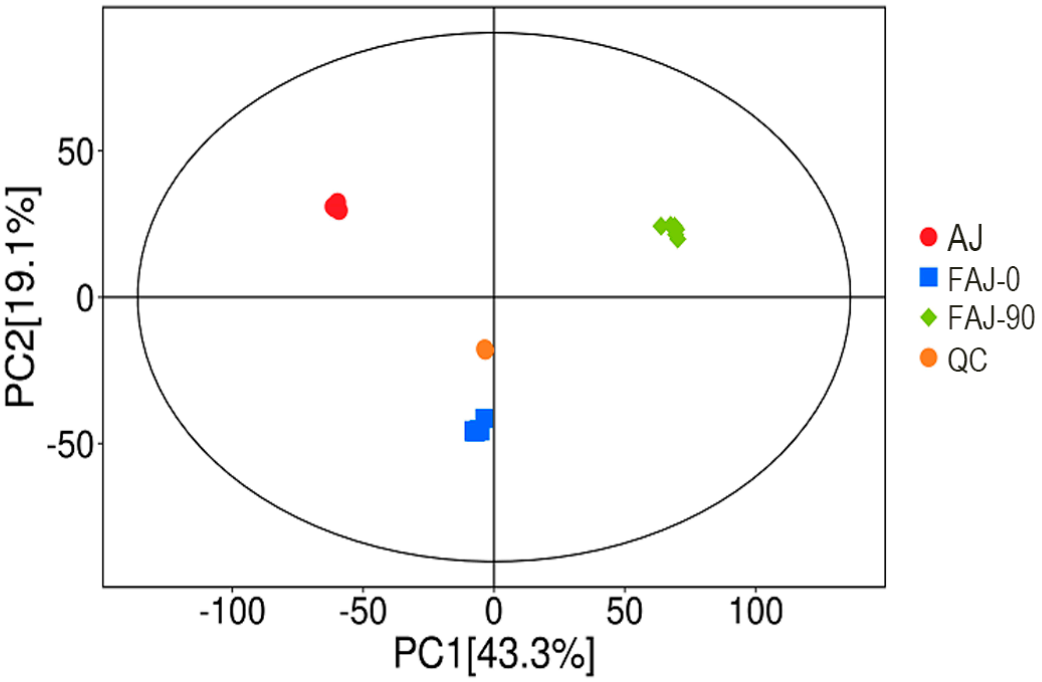

Figure S1. Principal component analysis (PCA) score plot of metabolites in aronia juice during fermentation and storage. AJ represents the unfermented aronia juice (control); FAJ-0 and FAJ-90 represent fermented aronia juice stored for 0 and 90 days, respectively; QC, quality control sample.

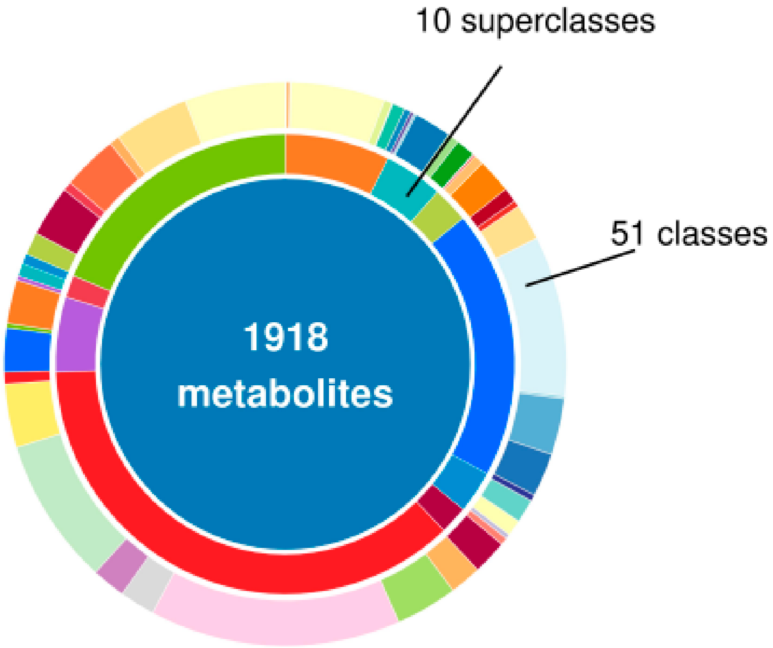

Figure S2. Classification of all detected metabolites. A total of 1918 metabolites were annotated and classified into 10 superclasses, which were further divided into 51 classes.

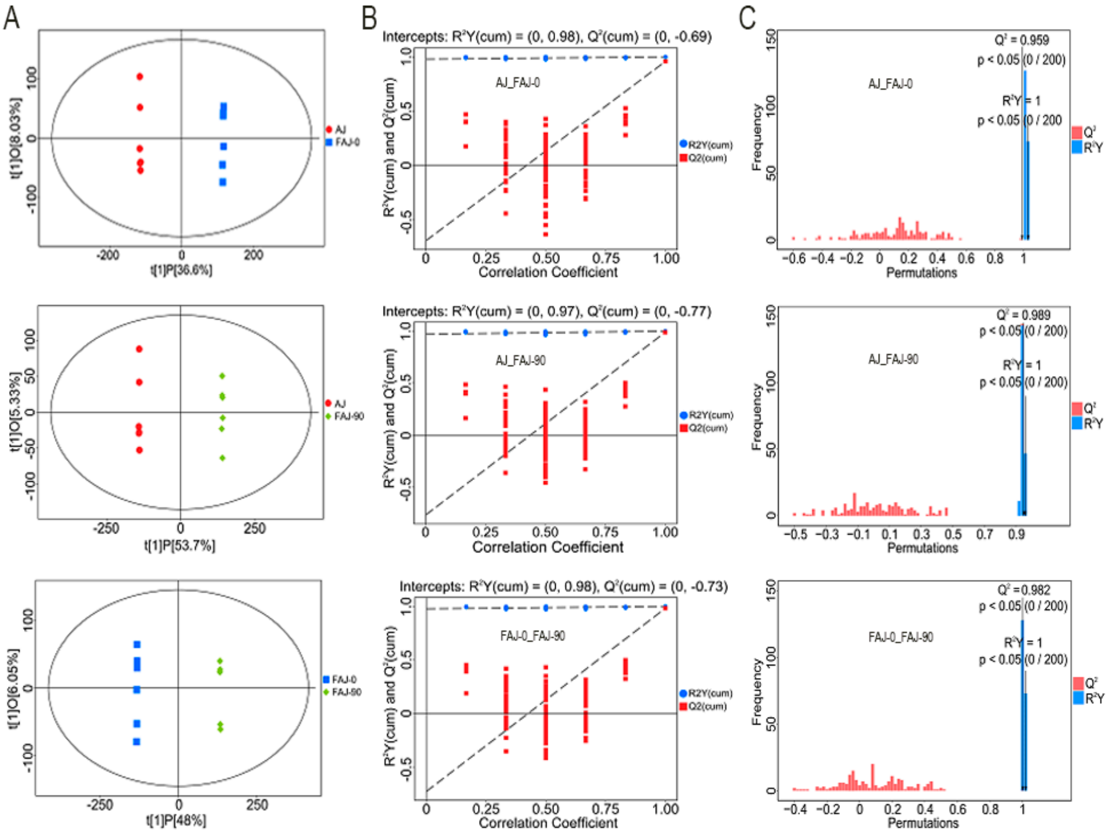

Figure S3. Orthogonal partial least squares-discriminant analysis (OPLS-DA) of metabolomics data between groups. (A) OPLS-DA score plots; (B) Permutation test plots of OPLS-DA models; (C) Histograms of 200-permutation tests. From top to bottom, the plots represent comparisons of AJ vs. FAJ-0, AJ vs. FAJ-90, and FAJ-0 vs. FAJ-90, respectively. AJ, unfermented Aronia melanocarpa juice (control); FAJ-0, fermented Aronia melanocarpa juice at day 0; FAJ-90, fermented Aronia melanocarpa juice stored for 90 days.

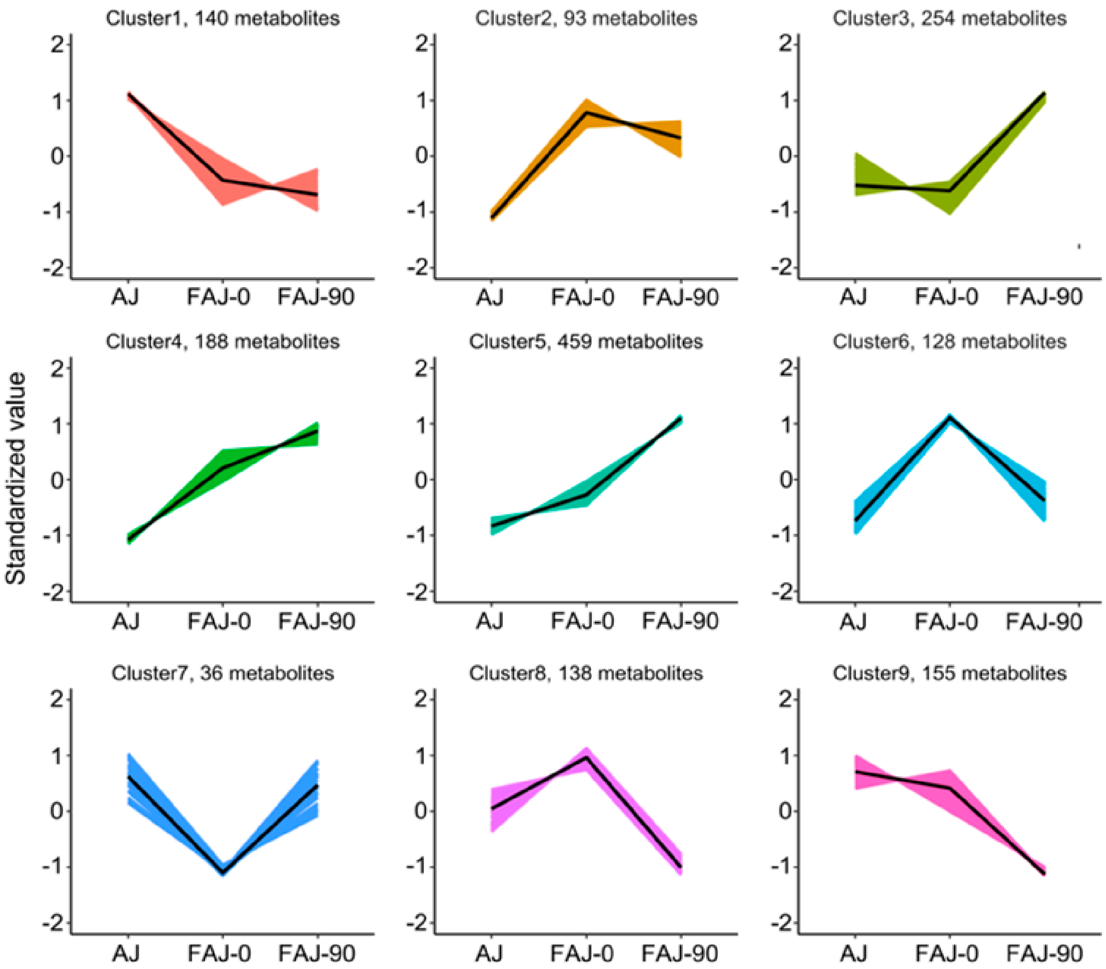

Figure S4. Expression pattern clustering analysis of differentially expressed metabolites. The differentially expressed metabolites were grouped into 9 clusters (Cluster 1–9) based on their expression profiles. Each cluster shows the standardized expression trend of the corresponding metabolites in the AJ, FAJ-0, and FAJ-90 groups, with the number of metabolites in each cluster indicated in the title. AJ, unfermented Aronia melanocarpa juice (control); FAJ-0, fermented Aronia melanocarpa juice at day 0; FAJ-90, fermented Aronia melanocarpa juice stored for 90 days.

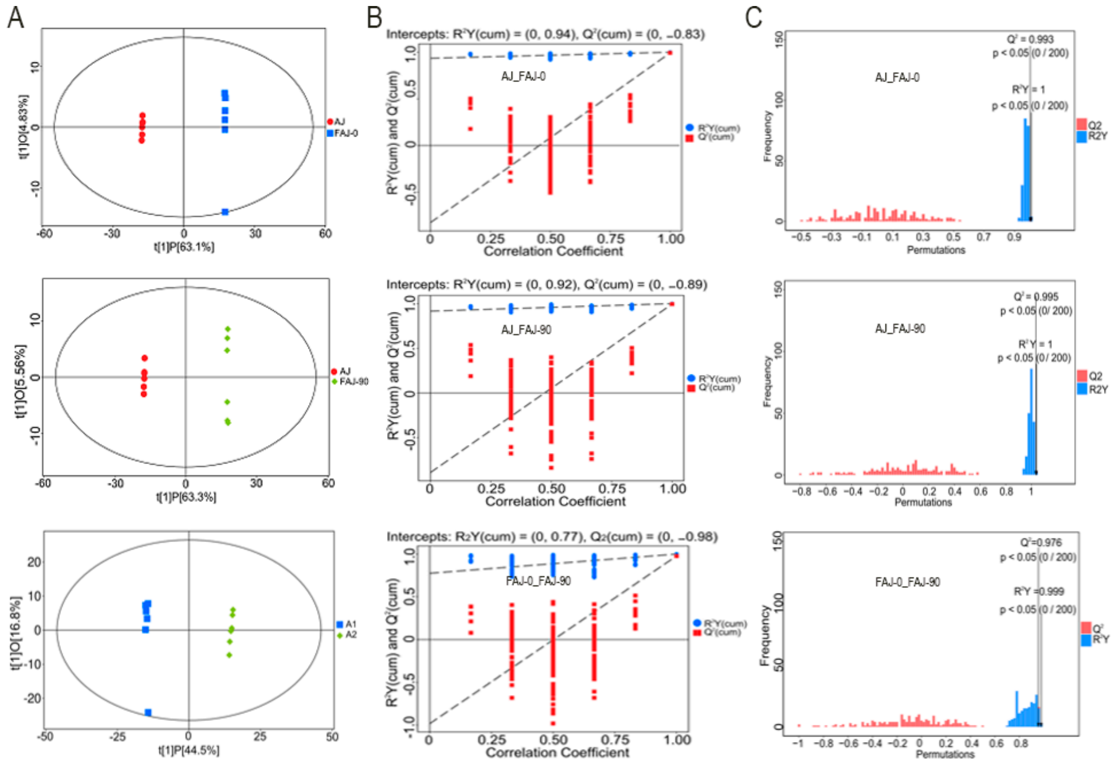

Figure S5. Orthogonal partial least squares-discriminant analysis (OPLS-DA) of volatile metabolites in three groups of Aronia melanocarpa juice. (A) OPLS-DA score plots; (B) Permutation test plots of OPLS-DA models; (C) Histograms of 200-permutation tests. From top to bottom, the plots represent comparisons of AJ vs. FAJ-0, AJ vs. FAJ-90, and FAJ-0 vs. FAJ-90, respectively. AJ, unfermented Aronia melanocarpa juice (control); FAJ-0, fermented Aronia melanocarpa juice at day 0; FAJ-90, fermented Aronia melanocarpa juice stored for 90 days.

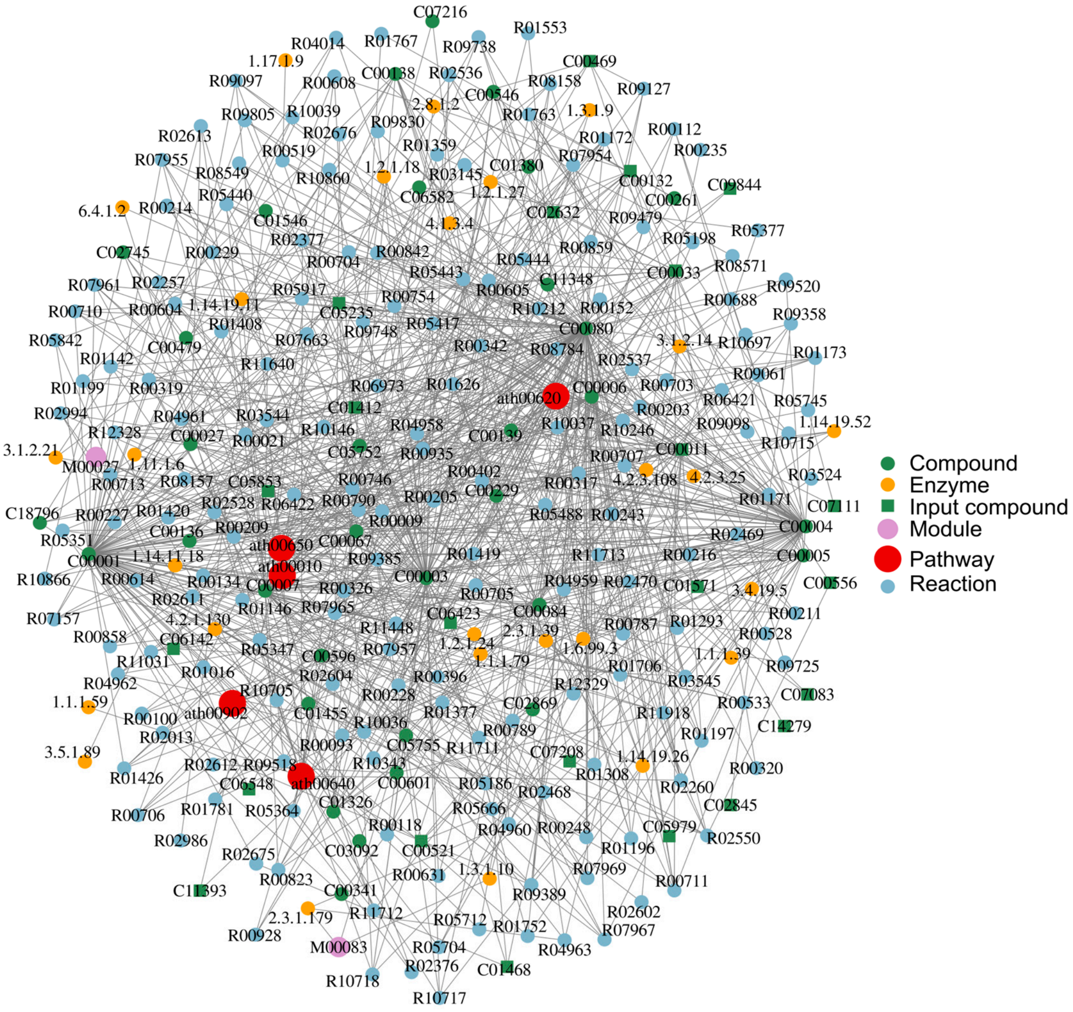

Figure S6. Interaction network of differential metabolites, reactions and pathways. Nodes represent compounds, enzymes, input compounds, modules, and pathways, and the lines represent the metabolic reaction relationships.
